# Relations of neuropeptide Y and heme oxygenase-1 expressions with fetal brain injury in rats with intrahepatic cholestasis of pregnancy^1^

**DOI:** 10.1590/s0102-865020190040000001

**Published:** 2019-05-06

**Authors:** Hongxia Li, Bofeng Liu, Chunyan Gu, Xiao Zeng, Yali Liu, Susu Zhang, Haiye Gong, Yong Shao, Zhenwei Yao, Ruifang An

**Affiliations:** I Doctor, Department of Obstetrics and Gynecology, The First Affiliated Hospital, Xi’an Jiao Tong University; and Department of Gynecology, Affiliated Hospital of Yan’an University, China. Design of the study, acquisition of data, technical procedures, final approval.; II Master, Department of Anatomy, Yan’an University Medical College, China. Acquisition of data, technical procedures, manuscript preparation, final approval.; III Bachelor, Department of Gynecology, Affiliated Hospital of Yan’an University, China. Acquisition of data, technical procedures, final approval.; IV Master, Affiliated Hospital of Yan’an University, China. Acquisition of data, technical procedures, final approval.; V Master, Department of Obstetrics and Gynecology, The First Affiliated Hospital, Chongqing Medical University, China. Statistical analysis, manuscript preparation, final approval.; VI Doctor, Department of Obstetrics and Gynecology, The First Affiliated Hospital, Xi’an Jiao Tong University, China. Design of the study, manuscript preparation, final approval.

**Keywords:** Cholestasis, Intrahepatic, Neuropeptide Y, Heme Oxygenase-1, Brain, Rats

## Abstract

**Purpose:**

To investigate the relations of neuropeptide Y (NPY) and heme oxygenase-1 (HO-1) expressions with fetal brain injury in rats with intrahepatic cholestasis of pregnancy (ICP).

**Methods:**

Sixty rats pregnant for 15 days were randomly divided into experimental and control groups. The ICP model was established in experimental group. On the 21st day, the blood biochemical test, histopathological examination of pregnant rat liver and fetal brain tissues and immunohistochemical analysis of fetal rat brain tissues were performed.

**Results:**

On the 21st day, the alanineaminotransferase, aspartate aminotransferase and total bile acid levels in experimental group were significantly higher than control group (P<0.01). Compared with control group, there was obvious vacuolar degeneration in pregnant rat liver tissue and fetal brain tissue in experimental group. NPY expression in fetal brain tissue was negative in control group and positive in experimental group. HO-1 expression in fetal brain tissue was strongly positive in control group and positive in experimental group. There was significant difference of immunohistochemical staining optical density between two groups (P<0.01).

**Conclusion:**

In fetal brain of ICP rats, the NPY expression is increased, and the HO-1 expression is decreased, which may be related to the fetal brain injury.

## Introduction

 Intrahepatic cholestasis of pregnancy (ICP) is a special complication of pregnancy, which occurs in the middle and late stage of pregnancy. The main clinical manifestations of ICP include pruritus, jaundice, elevated serum bile acid level and slight elevation of hepatic transaminase^1^. The prognosis of pregnant women with ICP is good, but the fetuses are prone to premature delivery, distress, asphyxia, central nervous system injury, and even intrauterine fetal death, which lead to the significantly increased perinatal mortality rate and caesarean section rate^2^. At present, the etiology and pathogenesis of ICP are not very clear. They are mainly related to estrogen factors, family genetic factors, and environmental factors^3^ ^,^ ^4^. With the development of neuroendocrine immunology, some scholars have paid more and more attention to the role of neuroendocrine immunity in the process of ICP. Neuropeptide Y (NPY) is a polypeptide composed of 36 amino acids. It acts as the neurotransmitter, and plays a role in nerve regulation and nerve secretion^5^. NPY is widely distributed in the tissues of body, especially in the nervous system. It is related to the brain injury, cerebral apoplexy and neonatal hypoxic-ischemic encephalopathy (HIE)^6^. Heme oxygenase (HO) is a family of microsomal enzymes with high conservatism in evolution. It has important function in regulation of iron ion stability and antioxidant defense^7^. The metabolite of HO, carbon monoxide, acts as a gas neurotransmitter and plays an important role in vascular dilation^8^. There are three isozymes of HO, including HO-1, HO-2 and HO-3. HO-1 is an inducible type, and is widely distributed in the spleen, liver, bone marrow and other tissues. The current studies are focuses on HO-1^9^ ^,^ ^10^. It is found that, HO-1 is related to the hypertensive disorder complicating pregnancy and intrauterine growth retardation^11^. Until now the relations of NPY and HO-1 with ICP is seldom reported. This study established a pregnant rat model of ICP, and investigated the relations of NPY and HO-1 expressions with fetal brain injury in rats with ICP. The objective was to provide one more theoretical basis for further studying the etiology of ICP and its clinical treatment.

## Methods

 This study was carried out in strict accordance with the recommendations in the Guide for the Care and Use of Laboratory Animals of the National Institutes of Health. The animal use protocol has been reviewed and approved by the Institutional Animal Care and Use Committee (IACUC) of The First Affiliated Hospital of Xi’an Jiao Tong University.

### Animals and grouping

 A total of 60 female Sprague-Dawley rats (5-6 months of age; 200-250 g; provided by Laboratory Animal Centre, The Third Minitary Medical University, Chongqing, China) were raised in a barrier system (avoiding strong light and noise; room temperature; relative humidity 60%-70%; 12/12-h day-night cycle; free to feed and water). In the estrus, the female rats were raised with male SD rats (5-6 months of age; 200-250 g; provided by Laboratory Animal Centre, The Third Minitary Medical University, Chongqing, China) in one cage, with male/female ratio of 4: 1. The shedding of the vaginal plug was observed every day. The day of vaginal plug shedding was defined as the first day of pregnancy. The rats were raised to the 15th day of pregnancy. Then the pregnant rats were randomly divided into experimental group and control group, 30 rats in each group.

### Establishment of ICP model

 ICP model of rats was established according to the reported methods^12^ ^,^ ^13^. In the experimental group, 75 mg/kg progesterone (Sigma-Aldrich Corp., MO, US) and 1.25 mg/kg 17-α-ethinyl estradiol (Sigma-Aldrich Corp., MO, US) were injected subcutaneously at the medial hind limb, once every day from the 15th day of pregnancy. In control group, the 2.5 ml/kg refined oil (Chongqing Chemical Reagent Co., Ltd., Chongqing, China) was injected subcutaneously at the medial limb at the time the same with experimental group.

### Determination of blood biochemical indexes

 At the 21st day of pregnancy, 1 ml of blood was taken from the orbital vein, and the blood biochemical indexes including alanineaminotransferase (ALT), aspartate aminotransferase (AST) and total bile acid (TBA) were determined using TBA-2000FR automatic biochemical analyzer (Toshiba Medical Systems China Co., Ltd., Beijing, China).

### Histopathological examination

 Thirty pregnancy rats were sacrificed by twisting neck. The pregnant rat liver and fetal brain tissues were taken and fixed immediately using 4% paraformaldehyde. After paraffin embedding and hematoxylin-eosin staining, the histopathological changes were observed under DVM6 optical microscope (Leica Science Lab, Leica Camera AG Berlin, Germany).

### Immunohistochemical staining

 The paraffin sections of fetal rat brain tissues were prepared. 2 μm paraffin sections were deparaffinized, and then washed with distilled water. After soaking in PBS for 5 min, they were incubated with 3% H_2_O_2_ at room temperature for 15 min. After washing with PBS, the appropriate proportion of primary antibodies (rabbit anti-rat NPY, rabbit anti-rat HO-1; Wuhan Boster Bioengineering Co., Ltd., Wuhan, China) were added for incubation at 37^o^C for 1 h, followed by washing with PBS. Then the biotinylated secondary antibody (HRP-labeled goat anti-rabbit IgG, Beijing Zhongshan Golden Bridge Biotechnology Co., Ltd., Beijing, China) was added, followed by incubation at 37^o^C for 30 min. After washing with PBS, the DAB (Wuhan Boster Bioengineering Co., Ltd., Wuhan, China) staining was performed, followed by washing with water, restaining, dehydration and mounting. The semi-quantitative analysis was performed with 801 image analysis system (Jiangsu Jieda Technology Development Co., Ltd., Nanjing, China). Two sections were randomly selected from each group, and 5 visual fields of each section were selected. The staining intensity was presented using average optical density (OD).

### Statistical analysis

 All statistical analysis was carried out using SPSS22.0 software (SPSS Inc., Chicago, IL, USA). The measurement data were presented as mean±SD, and were compared using t test. P<0.05 was considered as statistically significant.

## Results

### Biochemical indexes of pregnant rats in two groups

 As shown in Table 1, at the 21st day of pregnancy, the levels of ALT, AST and TBA in control group were 42.91±9.66 IU/L, 49.11±11.47 IU/L and 24.15±3.32 μmol/L, respectively, and those in experimental group were 228.95±9.02 IU/L, 432.58±21.12 IU/L and 78.37±6.18 μmol/L, respectively. There was significant difference of each index between two groups (P<0.01). This indicated that, the ICP model of rats was successfully established. 

**Table 1:** Biochemical indicators after inducing model in two group.

Group	n	ALT (IU/L)	AST (IU/L)	TBA (μmol/L)
Control	30	42.91±9.66	49.11±11.47	24.15±3.32
Experimental	30	228.95±9.02^*^	432.58±21.12^*^	78.37±6.18^*^

^*^P < 0.01 compared with control group. ALT, alanineaminotransferase; AST, aspartate aminotransferase; TBA, total bile acid.

### Histopathological results of liver tissue in pregnant rats

**Figure 1:**
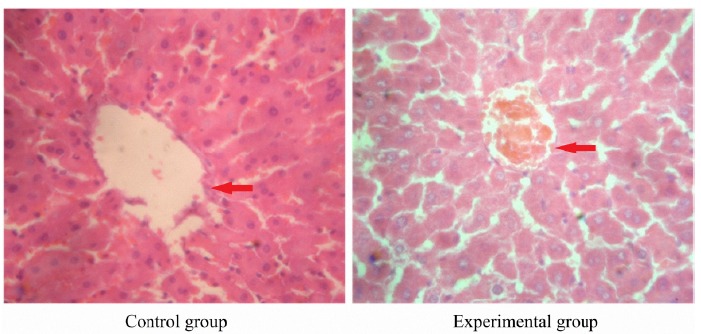
Histopathological results of liver tissue in pregnant rats in control group and experimental group (hematoxylin-eosin staining, ×200). Control group: there was no obvious morphological change in liver tissue of pregnant rats. Experimental group: the partial liver cells presented granular degeneration and vacuolar degeneration.

 In control group, there was no obvious morphological change in liver tissue of pregnant rats. In the experimental group, partial liver cells presented granular degeneration and vacuolar degeneration. There were bile plugs in partial capillary bile ducts. The structure of hepatic lobules was normal (Fig. 1).

### Histopathological results of brain tissue in fetal rats

**Figure 2:**
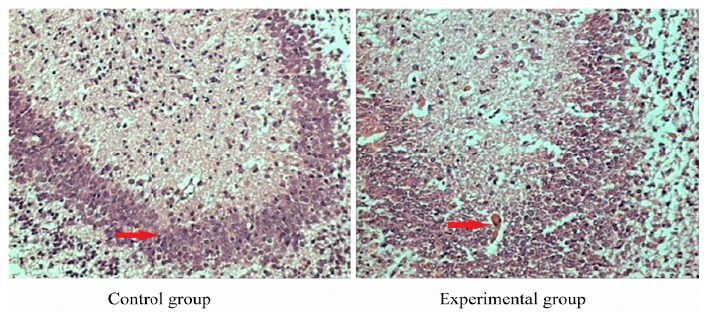
Histopathological results of fetal brain tissue in control group and experimental group (hematoxylin-eosin staining, ×200). Control group: no edema was observed in brain tissue of fetal rats. The cell and intercellular structure were normal. Experimental group: the brain tissue was loose, and the vacuolar degeneration was obvious. Parts of the cells were lysised, and even disappeared.

 In control group, no edema was observed in brain tissue of fetal rats. The cell and intercellular structure were normal. In experimental group, the brain tissue was loose, and the vacuolar degeneration was obvious. The parts of the cells were lysised, and even disappeared (Fig. 2).

### Immunohistochemical results of NPY in fetal brain tissue

**Figure 3:**
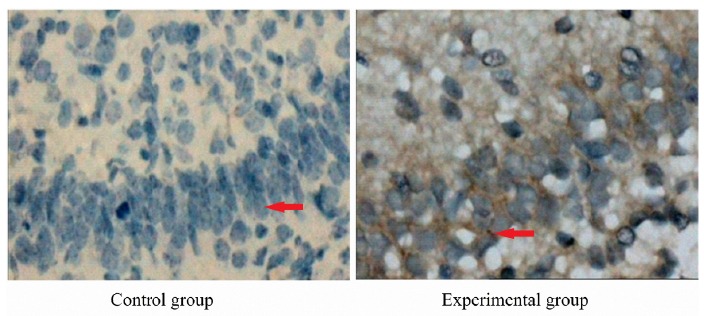
Negative neuropeptide Y expression in fetal brain tissue in control group and positive neuropeptide Y expression in fetal brain tissue in experimental group (×400). Control group: there were partial of cell membrane coloration in the hippocampus of fetal brain tissues, without positive neuropeptide Y-immunoreactive substance. Experimental group: the positive neuropeptide Y-immunoreactive neurons were mainly located in the hippocampus, which presented brown. The positive immunoreactive granules were mainly distributed in the cytoplasm and surface of the cell membrane.

 In control group, there were partial of cell membrane coloration in the hippocampus of fetal brain tissues, without positive NPY-immunoreactive substance. In the experimental group, the positive NPY immunoreactive neurons were mainly located in the hippocampus, which presented brown. The positive immunoreactive granules were mainly distributed in the cytoplasm and surface of the cell membrane (Fig. 3).

### Immunohistochemical results of HO-1 in fetal brain tissue

**Figure 4:**
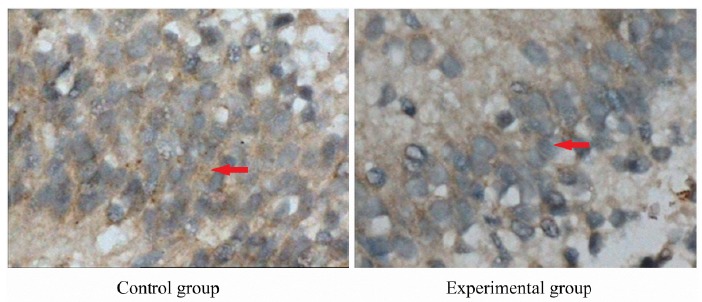
Strongly positive heme oxygenase-1 expression in fetal brain tissue in control group and positive heme oxygenase-1 expression in fetal brain tissue in experimental group (×400). Control group: the heme oxygenase-1 immune-positive cells in hippocampal CAl region of fetal brain were stained brown, which were mainly distributed in the cytoplasm and cell membrane surface, presenting strongly positive. Experimental group: there were less heme oxygenase-1 immune-positive cells in hippocampal CAl region, which were stained yellow, presenting positive.

 In control group, the HO-1 immune-positive cells in hippocampal CAl region of fetal brain were stained brown, which were mainly distributed in the cytoplasm and cell membrane surface, presenting strongly positive. In the experimental group, there were less immune-positive cells in hippocampal CAl region, which were stained yellow, presenting positive (Fig. 4).

### Image analysis results of immunohistochemical staining

 Results of image analysis showed that, the OD value of NPY immunohistochemical staining in experimental group was 1.08425±0.0179, which was significantly higher than 0.6324±0.0246 in control group (P<0.01). The OD value of HO-1 immunohistochemical staining in experimental group was 0.8876±0.0208, which was significantly higher than 1.638±0.0271 in control group (P<0.01) (Table 2).

**Table 2:** Image analysis results of immunohistochemical staining in two groups (OD).

Group	n	NPY	HO-1
Control	30	0.6324±0.0246	1.638±0.0271
Experimental	30	1.08425±0.0179^*^	0.8876±0.0208^*^

^*^P<0.01 compared with control group. OD, optical density; NPY, neuropeptide Y; HO-1, heme oxygenase-1. Control group. Experimental group.

## Discussion

 It is reported that, the liver biochemical and pathological changes of pregnant rats after treatment with estrogen are similar to those of human with ICP, and this can be used as an animal model for studying human ICP. However, it is found that the ICP model established by this method shows slight necrosis in the liver under light microscope, which is not consistent with the fact that there is no spotty necrosis change in human ICP^14^. This suggests that the establishment method of ICP model using estrogen alone is not perfect. Valleio *et al.* ^15^ find that, pregnant women with oral progesterone present the ICP symptoms including itching. Therefore, in some scholars’ researches, estrogen and progesterone are combined to establish the ICP animal model, and the liver biochemical and pathological changes of animal are very similar to those of human ICP^16^. In this study, estrogen and progestogen were combined to establish the ICP model of pregnant rats. The blood biochemical test showed that, at the 21st day of pregnancy, the levels of ALT, AST and TBA in the experimental group were significantly higher than those in the control group, respectively (P<0.01). In addition, the liver histological examination showed that, in control group, there was no obvious morphological change in liver. In the experimental group, partial liver cells presented granular degeneration and vacuolar degeneration. There were bile plugs in partial capillary bile ducts. The structure of hepatic lobules was normal. This indicates that, the ICP model of pregnant rats has been successfully established. 

 Animal clinical experiments have found that, NPY is associated with brain damage, stroke, and neonatal HIE^17^ ^-^ ^19^. HIE refers to the brain injury caused by reduced oxygen supply reduction and/or reduced blood supply secondary to late pregnancy, delivery or neonatal period. It is the main form of perinatal brain injury, and is the main cause of acute death^19^. Previous study^20^ finds that, in HIE patients the plasma NPY level is increased significantly. The higher the HIE level is, the higher the plasma NPY level is. The NPY level in the recovery phase is decreased significantly. This suggests that, the plasma NPY is involved in the pathophysiological process of HIE. In neonatal asphyxia, the plasma NPY is significantly increased, resulting in the increase of cerebral vascular resistance and the decrease of cerebral blood flow. This becomes one of the causes of HIE^6^. NPY can inhibit the vasodilation effect of blood vessels on adenosine, acetylcholine and other vasodilator substances. This causes the accommodative disorder of cerebral vasodilatation and contraction function, leading to further contraction of cerebral blood vessels and reduction of cerebral blood flow^21^. In addition, NPY can promote the hydrolysis of phosphatidylinositol on the cell membrane, producing two diacylglycerol and inositol triphosphate (IP3). As a second messenger, IP3 can promote the opening of Ca^2+^ channels in the endoplasmic reticulum and increase the intracellular Ca^2+^ concentration, thus aggravating hypoxic ischemic brain damage^22^. The results of this study showed that, NPY was mainly distributed in the hippocampus of ICP fetal rats. The expression of NPY in the experimental group was increased significantly, compared with control group. This suggests that, NPY may be related to fetal distress in ICP.

 Recent study has shown that, HO-1 has the anti-inflammatory, anti-apoptotic and anti-proliferative effects, and plays a cytoprotective role in related diseases such as arteriosclerosis, cerebral ischemia and organ transplant rejection. Therefore, the biological activity of HO-1 and its protective effect in many clinical diseases have attracted the attention of many scholars. It is found that, the antioxidant role of HO-1 has the best expression threshold^23^. The moderate expression HO-1 can reduce the cell death, protein oxidation and lipid peroxidation, thus promoting the cell proliferation. The over expression of HO-1 may damage the integrity of cells. The low expression of HO-1 leads to the decreased protective effect, thus the cells are susceptible to the invasion of harmful factors^24^. In this study, the expression of HO-1 in the brain tissue of fetal rats was detected. Results showed that, the immune staining of HO-1 in the hippocampus was obvious in the control group, while it was weakened the experimental group. Because the lesion degree of ICP is different, the expression of HO-1 is different. The more severe the lesion is, the weaker the HO-1 expression is, indicating its weaker protective effect. The study in which the transgenic mice are used as experiment materials proves that, the transgenic mice with over expression of HO-1 present significantly reduced infarction focus in brain ischemic injury and permanent focal cerebral ischemia injury. This indicates that, the expression of HO-1 in the ischemia and hypoxia stress state is related to the self protection mechanism of body^9^ ^-^ ^10^. Therefore, it is speculated that, the abnormal expression of HO-1 is one of the causes of poor prognosis of fetus in ICP.

## Conclusions

 In fetal brain of ICP rats, the NPY expression is increased, and the HO-1 expression is decreased, which may be related to the fetal brain injury. This study has provided one more theoretical basis for further studying the etiology of ICP and its clinical treatment. However, whether there are other mechanisms of ICP should be further studied.
